# Cross-Reactivity and Functionality of Approved Human Immune Checkpoint Blockers in Dogs

**DOI:** 10.3390/cancers13040785

**Published:** 2021-02-13

**Authors:** Stanislav Pantelyushin, Elisabeth Ranninger, Diego Guerrera, Gregor Hutter, Caroline Maake, Enni Markkanen, Regula Bettschart-Wolfensberger, Carla Rohrer Bley, Heinz Läubli, Johannes vom Berg

**Affiliations:** 1 Institute of Laboratory Animal Science, University of Zurich, CH-8952 Schlieren, Switzerland; stanislav.pantelyushin@uzh.ch (S.P.); diego.guerrera@gmail.com (D.G.); 2 Institute of Anatomy, University of Zurich, CH-8057 Zurich, Switzerland; caroline.maake@anatomy.uzh.ch; 3 Department of Clinical and Diagnostic Services, Section of Anesthesiology, Vetsuisse Faculty, University of Zurich, CH-8057 Zurich, Switzerland; eranninger@vetclinics.uzh.ch (E.R.); rbettschart@vetclinics.uzh.ch (R.B.-W.); 4 Department of Biomedicine, University of Basel, CH-4031 Basel, Switzerland; gregor.hutter@usb.ch (G.H.); heinz.laeubli@unibas.ch (H.L.); 5 Department of Neurosurgery, University Hospital Basel, CH-4031 Basel, Switzerland; 6 Institute of Veterinary Pharmacology and Toxicology, Vetsuisse Faculty, University of Zurich, CH-8057 Zurich, Switzerland; enni.markkanen@vetpharm.uzh.ch; 7 Division of Radiation Oncology, Vetsuisse Faculty, University of Zurich, CH-8057 Zurich, Switzerland; crohrer@vetclinics.uzh.ch; 8 Division of Medical Oncology, University Hospital Basel, CH-4031 Basel, Switzerland

**Keywords:** translational cancer research, canine cancer, veterinary oncology, T cells, checkpoint blockade, PD-1/PD-L1, cancer immunotherapy, combination therapy, response biomarker, immunophenotyping

## Abstract

**Simple Summary:**

Immunotherapy with immune checkpoint inhibitors (ICI) has been a major advance in the management of cancers. To increase response rates, numerous new cancer immunotherapies are under development, to be used as monotherapies or as combination with existing ICIs. Lately, companion dogs suffering from neoplastic diseases have gained recognition for the development of cancer drugs. Despite evidence for PD-1/PD-L1 blockade eliciting anti-tumor responses, there are currently no commercially available ICIs for use in dogs. Here, we explored the potential use of seven FDA-approved human ICIs in dogs. Atezolizumab is cross-reactive, blocks PD-1/PD-L1 interaction and increases cytokine production of T cells derived from healthy donors and dog cancer patients in vitro. Response to atezolizumab seems to be dependent on the composition of blood lymphocytes and tumor type. Our data provides a rationale for testing promising treatment modalities and combination therapies involving PD-1/PD-L1 blockade in dog trials to advance veterinary and human medicine.

**Abstract:**

Background: Rodent cancer models have limitations in predicting efficacy, tolerability and accompanying biomarkers of ICIs in humans. Companion dogs suffering from neoplastic diseases have gained attention as a highly relevant translational disease model. Despite successful reports of PD-1/PD-L1 blockade in dogs, no compounds are available for veterinary medicine. Methods: Here, we assessed suitability of seven FDA-approved human ICIs to target CTLA-4 or PD-1/PD-L1 in dogs. Cross-reactivity and blocking potential was assessed using ELISA and flow cytometry. Functional responses were assessed on peripheral blood mononuclear cells (PBMCs) derived from healthy donors (*n* = 12) and cancer patient dogs (*n* = 27) as cytokine production after stimulation. Immune composition and target expression of healthy donors and cancer patients was assessed via flow cytometry. Results: Four candidates showed cross-reactivity and two blocked the interaction of canine PD-1 and PD-L1. Of those, only atezolizumab significantly increased cytokine production of healthy and patient derived PBMCs in vitro. Especially lymphoma patient PBMCs responded with increased cytokine production. In other types of cancer, response to atezolizumab appeared to correlate with a lower frequency of CD8 T cells. Conclusions: Cross-functionality of atezolizumab encourages reverse translational efforts using (combination) immunotherapies in companion dog tumor patients to benefit both veterinary and human medicine.

## 1. Introduction

The development and implementation of immune checkpoint inhibitors (ICIs) have profoundly changed our view of effective anti-cancer treatments. Current FDA-approved ICIs target two pathways with three main targets, namely Cytotoxic T-Lymphocyte Associated Protein 4 (CTLA-4), and Programmed Death 1 and its Ligand 1 (PD-1/PD-L1). To date, seven monoclonal antibodies blocking those molecules have been approved for various conditions, alone or in combinations. These include ipilimumab (anti-CTLA4), nivolumab, pembrolizumab and cemiplimab (anti-PD-1), as well as atezolizumab, avelumab and durvalumab (anti-PD-L1) [1]; and many more are in late stages of clinical development [2].

Despite the encouraging success of ICIs in a growing number of indications, various cancers do not respond to current strategies [1]. As new immunotherapies, their combinations and various treatment regimens are being implemented and tested at an increasing pace [1], the numbers of eligible patients that satisfy stringent clinical trial inclusion criteria could become scarce [3].

Inbred mouse models allow critical insights regarding initial efficacy and mechanism of action, but also vastly underrepresent the heterogeneity and complex interplay of human immune cells and cancers. Companion dogs can provide a translational model to complement studies in mice. They naturally develop a broad spectrum of cancers, which have profound similarities with human diseases [4,5]. Other important parallels include the similar living environments and hence ‘exposomes’ [6], as well as the spontaneous nature of cancer development. The shorter lifespan of dogs also means they develop and succumb to cancer earlier than humans. This provides opportunities to pre-evaluate promising treatments faster than in clinical human cancer trials and makes dogs a very attractive translational model for cancer immunotherapy [5].

Targeting the PD-1/PD-L1 axis has become the first choice for combination therapies in human clinical testing [3]. Most dog studies on ICIs investigated this axis and a number of attempts have been made to develop blocking antibodies specific for dogs [7,8,9,10]. These pioneering findings have showcased the importance of this axis also in dogs by studying peripheral blood mononuclear cells (PBMCs) from healthy and tumor-bearing dogs [7,11,12], tumor explant cultures [9] and tumor cell lines [8,10,13]. Moreover, increased expression of PD-1 has been shown on canine T cells [9,12,14], as well as PD-L1 on canine antigen presenting cells (APCs) [10,12], and tumor cells [10,12,13,15]. However, to our knowledge, only two interventional studies in canine tumor patients have been published to date, showing some degree of success with anti-PD-L1 and anti-PD-1 antibodies [16,17]. Despite these promising results, no canine-specific ICIs for use in veterinary practice are commercially available. It is relevant as it is challenging—mostly for cost reasons—to develop canine specific ICIs and dog owners are rarely in a position to pay the high price for immunotherapy [18]. Additionally, well-characterized approved human antibodies generally have superior biophysical properties and ‘developability’, which significantly improves their success as a potent therapeutic [19,20]. Testing their functionality in dogs could provide a rationale for new (combination-) treatment regimens along the translational path from mouse to humans and for adaptation or direct use in veterinary oncology [21]. It is however unclear, which human ICIs could be effective in the therapy of canine cancers and whether there are prognostic markers to predict treatment response to ICI(s) in dogs.

Here, we aimed to bridge this gap and to provide a rationale for future translational and reverse-translational endeavors for immunotherapies in dogs using ICIs approved for treatment of human patients. We systematically analyzed cross-reactivity and potential efficacy of current FDA-approved human ICIs in vitro using canine cell lines and PBMCs from healthy donors as well as cancer patient-dogs with a particular focus on the PD-1/PD-L1 axis. We adopted methods and readouts which have been previously employed for preclinical development of ICIs in humans that show improved T cell responses to various stimuli [22,23]. Furthermore, we also evaluated their ability to block the interaction of canine PD-1 and PD-L1 and analyzed their functionality on canine immune cells in vitro. Finally, we characterized PBMC composition of responding vs. non-responding cancer patient samples.

## 2. Materials and Methods

### 2.1. Human Immune Checkpoint Inhibitor (ICI) Antibodies

Clinically approved ICI antibodies were obtained by H. L. at Department of Medical Oncology, Department of Internal Medicine, University Hospital Basel (Basel, Switzerland). ICIs were aliquoted in protein Lobind tubes (0030 108.116, Eppendorf, Hamburg, Germany) and stored at −80 °C.

### 2.2. Canine and Human Cell Line Culture

The canine cells SCC1 (derived from a canine oral squamous cell carcinoma [24]), CoSCC (isolated from a gingival squamous cell carcinoma [24]) and DUS (dog uterine stromal cells that were immortalized using SV40Tag [25]) were obtained directly from the above research groups by Dr. Enni Markkanen who further characterized them [15]. U87 cells were verified and provided by Prof. Gregor Hutter (University Hospital Basel). Cells were tested for absence of mycoplasma contamination using a MycoAlert kit (LT07-318 Lonza, Basel, Switzerland) in conjunction with the MycoAlert Assay Control Set (LT07-518, Lonza). A ratio of <0.9 of the assay readout was interpreted as mycoplasma negative. All cell lines were cultured in complete medium containing high glucose DMEM (21013024, Thermo Fisher, Waltham, MA, USA), 10% FBS (P30-2602, Pan Biotech, Aidenbach, Germany), GlutaMAX (2 mM) (35050061, Thermo Fisher), penicillin-streptomycin (100 units, 15140122, Thermo Fisher). cPD-L1 expression was induced with 10 ng/mL of recombinant cIFN-γ (781-CG-050, Novus Biologicals, Littleton, CO, USA) for 48 h.

### 2.3. Canine Soluble Recombinant Proteins

Soluble canine PD-1 with a human fragment crystallized (Fc) IgG1 fusion tag (scPD-1Fc) (70109-D02H) or with fusion His-tag (scPD-1His) (70109-D08H), as well as soluble canine PD-L1 with a human IgG1 fusion tag (scPD-L1Fc) (70110-D02H) were all obtained from Sino Biological (Beijing, China).

### 2.4. Sequence Alignments of Canine and Human CTLA-4, PD-1 and PD-L1

Amino acid sequences were obtained from the protein repository of NCBI with the following accession numbers: human CTLA-4 (NP_005205.2), PD-1 (NP_005009.2), PD-L1 (AAI13735.1); canine CTLA-4 (NP_001003106.1), PD-1 (NP_001301026.1), PD-L1 (BAO74172.1). The obtained sequences were aligned using the sequence alignment tool in CLC Main Workbench software version 7.8.1 (Qiagen, Hilden, Germany).

### 2.5. Dog PBMC Isolation, Thawing, Stimulation and Suppression

Whole blood from canine healthy donors and cancer patients was diluted with DPBS at a ratio of 1:1, and layered over 6 mL of Ficoll^®^ Paque Plus (GE17-1440-02, Sigma, St. Louis, MO, USA) in V-bottom 15 mL tubes. The samples were centrifuged at 900 *g* for 25 min at room temperature with brakes at lowest setting. The PBMC layer was transferred to a clean V-bottom 15 mL tube and washed with DPBS. The cells were counted and resuspend in ice cold freezing medium (5:4:1 RPMI1640: FBS: DMSO) on ice at 2 × 10^6^ cells/mL. The samples were initially preserved in a freezing container at −80 °C for 2 days and then transferred to −150 °C for long-term storage.

For analysis or stimulation of cryopreserved PBMCs, thawed aliquots were slowly diluted in warm complete medium containing RPMI1640 (21875034, Thermo Fisher), 10% FBS (P30-2602, Pan Biotech), GlutaMAX (2 mM, 35050061, Thermo Fisher), penicillin-streptomycin (100 units, 15140122, Thermo Fisher), sodium pyruvate (1 mM, 11360070, Thermo Fisher), 5 mL of non-essential amino acids (NEAA, 0.1 mM, 11140050, Thermo Fisher) and 12.5 mL HEPES buffer (25 mM, 15630080, Thermo Fisher). PBMCs were washed with warm medium and used in the assays at described concentrations.

For the polyclonal stimulation of PBMCs and assessment of PD-1 expression 2 × 10^5^ healthy donor derived PBMCs were stimulated with 2.5 μg/mL of concanavalin A (C2010, Sigma) and incubated for 48 h.

For the assessment of functional activity of human ICIs on canine PBMCs, 2 × 10^5^ healthy donor or cancer patient PBMCs were distributed/well, stimulated with 50 ng/mL of staphylococcal enterotoxin B (BT202, Toxin Technology, Sarasota, FL, USA) on U-bottom 96-well plates (353077, Corning, New York, NY, USA) and incubated for 72 h. For extra suppression of stimulation PBMCs were cultured with 10 μg/mL of scPD-L1Fc [9]. To reverse the suppression 10 μg/mL of indicated ICIs were added. Responders (R) were defined as the samples with >5% increase in cIFN-γ production over durvalumab (control), while non-responders (NR) were defined as <5% increase or a decrease in cIFN-γ production. To establish if durvalumab can be used as a non-binding IgG1 isotype control for atezolizumab and avelumab it was tested against a commercially available human IgG1 isotype control (#BE0297, Bio X Cell, Lebanon, NH, USA).

### 2.6. ELISA and Binding Assays

All plate binding, blocking and ELISA assays were performed on high binding plates (3369, Corning). Canine IFN-γ was quantified using Canine IFN-γ ELISA development kit (HRP, 3113-1H-6, Mabtech, Nacka Strand, Sweden). cCTLA-4His and cPD-1His was quantified using Anti-His-Horseradish Peroxidase antibody (130-092-785, Milteny, Bergisch Gladbach, Germany). PD-1 ICIs were detected using biotinylated anti-human IgG mAb (clone MT78, 3850-6-250, 1:1000, Mabtech, Nacka Strand, Sweden) and detected with Streptavidin-HRP (3310-9-1000, 1:1000, Mabtech).

### 2.7. Blocking of cPD-1 and cPD-L1 Binding Assay

To evaluate the ability of ICIs to block PD-1/PD-L1 binding, a blocking assay was conducted on a microwell plate using cPD-1His (70109-D08H, Sino Biological, Beijing, China) and scPD-L1Fc (70110-D02H, Sino Biological) fusion proteins. The bottom of a microwell plate was coated with 20 μg/mL of scPD-L1 and blocked with PBS containing 0.1% of bovine serum albumin and 0.05% Tween20. For blocking the binding, we either added a range of concentrations (0, 2.5, 5, 10 μg/mL) of PD-L1 blocking ICIs to the plate or incubated with 20 μg/mL of cPD-1His preincubated with a range of concentrations of PD-1-blocking ICIs (0, 2.5, 5, 10 μg/mL) in Eppendorf tubes at RT for 1 h. After that 20 μg/mL of pure cPD-1His (PD-L1 ICIs) or PD-1 ICI blocking mixes were added and incubated on a plate for 2h [17]. Bound cPD-1 was detected with 1:1000 diluted His-tag specific antibody coupled to HRP (130-092-785, Milteny) according to the manual. cPD-1His binding or lack thereof was detected using TMB (3,3′,5,5;-tetramethylbenzidine) chromogen solution (002023, Thermo Fisher). Relative optic density (% OD) was calculated from the OD in comparison with that of control without ICIs using a Spark multimode microplate reader (Tecan, Männedorf, Switzerland).

### 2.8. Flow Cytometry

Cells were washed with DPBS (14190144, Thermo Fisher) and always stained in a PBS staining mix containing 1:500 diluted Fixable Live/Dead Cell staining (Zombie Aqua, 423102, Biolegend, San Diego, CA, USA). Other antibodies or proteins were added at mentioned concentrations/dilutions to the same staining mix. Cells were incubated with primary staining or directly labeled antibodies for 20 min in the dark. Following three washes with DPBS cells were either resuspended in DPBS and acquired or stained for 20 min in the dark with secondary antibodies, washed three times with DPBS and acquired.

Human ICIs were detected by flow cytometry using anti-human Fc PE labeled antibody (clone HP6017, 409304, Biolegend) at 1:200 dilution. Canine PBMC were analyzed with the panel previously described (Table 1) [26]. Ki67 AF700 staining was performed using Foxp3/Transcription Factor Staining Buffer Set (00-5523-00, Thermo Fisher) according to manufacturer’s instructions (clone SolA15, 56-5698-82, Thermo Fisher).

For the UMAP analysis at least 100,000 live cells were acquired and analyzed using FlowJo software (BD, Franklin Lakes, NJ, USA). Using the UMAP plugin, 1500 merged events from each sample were visualized for non-responders (NR, *n* = 6) or responders (R, *n* = 19) to atezolizumab and from healthy donor responders (HD, *n* = 11). Cells were colored according to the cluster they were assigned to using the FlowSOM algorithm and manual annotation. The heat map was plotted using median fluorescence intensity of each marker for each population normalized to a −2 to 2 range of 8 key lineage markers within the nine cellular clusters using Heatmapper web application [27].

All cell acquisitions were performed using BD LSR Fortessa II (BD) and analyzed using FlowJo software version 10.4 (BD).

### 2.9. Statistics and Curve Fitting

Graph plotting and statistical analyses were performed using GraphPad Prism version 5.0 software (GraphPad, San Diego, CA, USA). A *p* value of less than 0.05 was considered statistically significant. Curve fit was performed using the log agonist versus response in GraphPad Prism version 8.

## 3. Results

### 3.1. Evaluation of ICIs Binding to Canine PD-1

**Figure 1:**
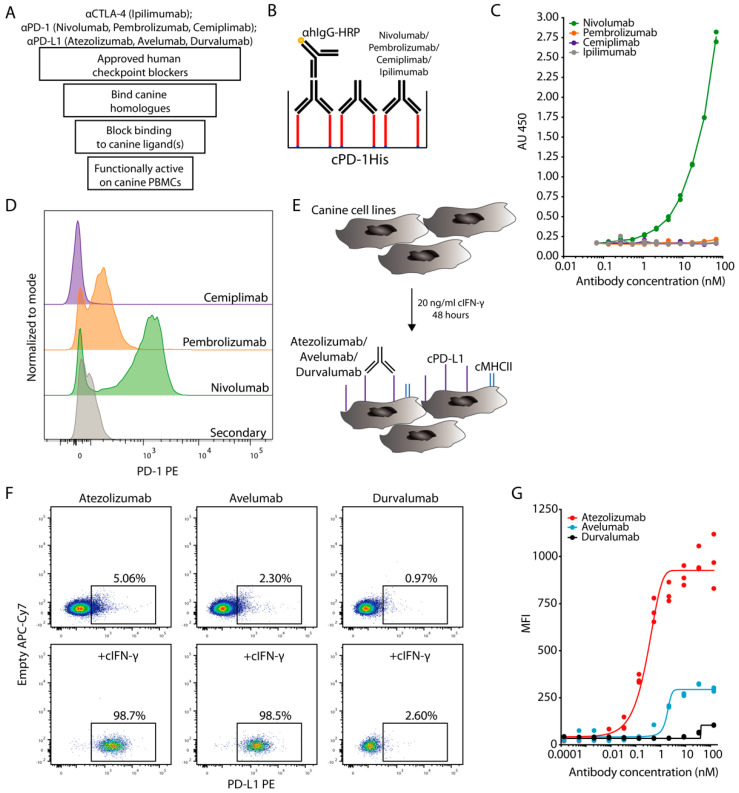
Cross-binding of human ICIs with canine homologues. (**A**) Schematic workflow for the evaluation steps of ICIs in dogs. (**B**,**C**) Plate-based assay to determine cPD-1 binding by nivolumab, pembrolizumab and cemiplimab was performed by coating 10 μg/mL of cPD-1His (red) on a microwell plate and incubation with various concentrations of respective ICIs, with ipilimumab acting as a negative control. Subsequently ICI binding was detected with anti-human IgG-HRP. (**B**) Schematic representation of the experiment, (**C**) Duplicates with fitted curve (log agonist versus response). Representative plot from two independent experiments. (**D**) Flow cytometric histograms show binding profiles of nivolumab, pembrolizumab and cemiplimab to canine T cells after 48 h of ConA stimulation. Pregated on Live Single CD45+CD5+ cells. Representative plots from 2 independent experiments (*n* = 3). (**E,F**) Flow cytometric evaluation of atezolizumab, avelumab and durvalumab binding cPD-L1 on canine SCC1 cell line stimulated with cIFN-γ. (**E**) Schematic representation of the experiment with upregulation of MHCII (blue) and cPD-L1 (purple) (**F**) Representative flow cytometric plots of cIFN-γ stimulated versus non-stimulated cells. Pregated on Live Single cells. (**G**) MFI binding curve comparison of atezolizumab, avelumab and durvalumab binding to cPD-L1. Triplicates with fitted curve (log agonist versus response). Representative plot from 3 independent experiments.

To circumvent the problem of reagent availability, we first developed a number of plate and cell-based binding assays, as well as functional in vitro assays with canine (c) PBMCs to test cross-reactivity of human ICIs. These were used to explore in a sequential approach if approved human ICIs bind, block binding interactions and provide functional benefits in dogs (Figure 1A). To establish the binding of nivolumab, pembrolizumab and cemiplimab to cPD-1, we bound cPD-1His to microplates and incubated it with increasing concentrations of nivolumab, pembrolizumab, cemiplimab and ipilimumab as a negative control (Figure 1B). Detection with a human IgG-specific antibody showed concentration dependent binding of nivolumab, while no significant binding could be detected for pembrolizumab, cemiplimab or ipilimumab (control) (Figure 1C).

We also tested the binding of the above human PD-1 blockers to cPBMCs stimulated with the mitogen concanavalin A (ConA). Since there are no commercially available agonistic canine-specific anti-CD3 antibodies, ConA is commonly used for polyclonal T cell stimulation in dogs [9]. Moreover, dog-specific anti-PD-1 or PD-L1 antibodies are also not widely available. Canine T cells have previously been shown to strongly upregulate PD-1 when assessed with specifically developed anti-canine PD-1 antibodies upon ConA activation [7]. Under these conditions we observed distinct PD-1 staining with nivolumab, while pembrolizumab provided only a small shift and staining was not detectable in case of cemiplimab by flow cytometry (Figure 1D). PD-1 is upregulated by activated T cells as a negative feedback regulator [9]. Accordingly, the staining with nivolumab was more pronounced on proliferating Ki67+ CD4+ and CD8+ T cells (Figure S1A,B). Overall, nivolumab consistently bound cPD-1, with pembrolizumab only weakly doing so at very high concentrations, while cemiplimab did not show cross-reactivity under any conditions.

### 3.2. Evaluation of ICIs Binding to Canine PD-L1

To test the binding of PD-L1-specific ICIs, we used the tumor cell lines squamous cell carcinoma 1 (SCC1), canine oral squamous cell carcinoma (CoSCC) and immortalized dog uterine stromal cell line (DUS), none of which appeared to express cPD-L1 under normal culture conditions. To assess the activation of Interferon (IFN)-γ activated site signaling [28], which also upregulates PD-L1 on canine tumor cell lines [10], we monitored major histocompatibility class II (MHCII) as a proxy for cIFN-γ signaling. We did not observe MHCII expression on any of the canine tumor cell lines at steady state, but its nearly complete upregulation on all three cell lines after 48 h of cIFN-γ stimulation (Figure 1E and Figure S1C).

Having confirmed cIFN-γ responsiveness, we assessed binding of atezolizumab, avelumab and durvalumab to these canine cell lines or a human glioma cell line that constitutively expresses high levels of PD-L1. Out of the three tested canine cell lines, the SCC1 cell line provided the most pronounced staining with atezolizumab and avelumab after stimulation with cIFN-γ, while no specific binding to canine cells was detected for durvalumab under any conditions (Figure 1F,G and Figure S1D,E). Of note, durvalumab binding to human PD-L1 was well detectable (Figure S1F). We concluded that out of the tested PD-L1 blockers atezolizumab and avelumab were able to bind to cPD-L1, while durvalumab did not.

### 3.3. Atezolizumab and Avelumab Block the Binding of Canine PD-1 and PD-L1

**Figure 2:**
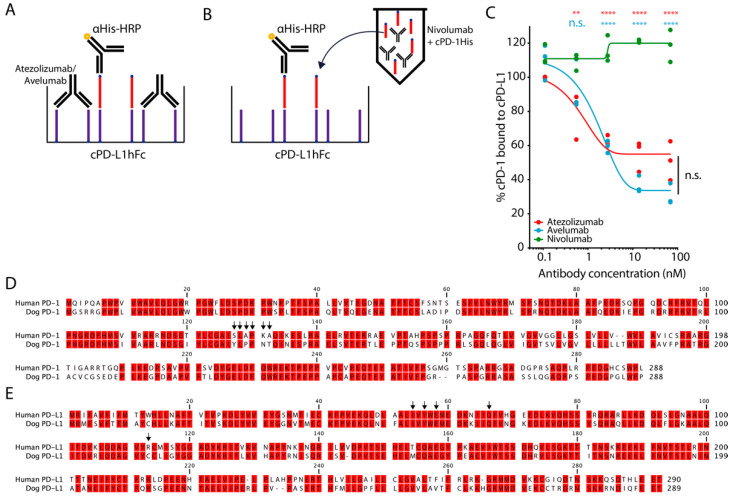
Blockade of interaction of PD-1 and PD-L1. (**A**,**B**) Outline of experimental setup blocking cPD-1/cPD-L1 binding by atezolizumab, avelumab and nivolumab. 20 μg/mL cPD-L1Fc (purple) was coated on a microwell plate and binding of 20 μg/mL cPD-1His (red) was detected on the plate, either (**A**) after blockade of cPD-L1 on the plate with atezolizumab and avelumab, or (**B**) preincubated with various concentrations of nivolumab [17] (**C**) Percentage of cPD-1 binding to cPD-L1 after blockade with indicated ICIs. Triplicates with fitted curve (log agonist versus response). Representative plot from three independent experiments (Mean, SEM, Two-way ANOVA with Bonferroni post-test comparing all pairs of columns, ** *p* < 0.01, **** *p* < 0.0001). (**D**,**E**) Sequence analysis of canine and human (**D**) PD-1 and (**E**) PD-L1. Black arrows depict crucial residues for binding each other and for blockade of the interaction by ICIs [29].

Subsequently, we tested whether the cross-reactive ICIs that target the PD-1/PD-L1 axis (nivolumab, atezolizumab and avelumab) blocked the binding of respective canine homologues. To this end, we coated a plate with cPD-L1Fc followed by incubation with various concentrations of atezolizumab and avelumab. In case of nivolumab, a range of concentrations was incubated in tubes with a fixed concentration of cPD-1His. Thereafter, the mixes with nivolumab were added to the wells with cPD-L1Fc, while the same concentration of cPD-1His was added to the wells with PD-L1 ICIs (Figure 2A,B) [17]. The quantification of bound cPD-1His revealed that only atezolizumab and avelumab blocked the binding of canine PD-1 and PD-L1, while nivolumab did not (Figure 2C).

The amino acid residues important for the binding of human PD-1 and PD-L1, the inhibitory mechanisms of their interactions by nivolumab, pembrolizumab, as well as atezolizumab, avelumab and durvalumab have been studied in depth [29]. The sequences of canine and human PD-1 and PD-L1 have been previously documented, compared and yielded 75.7% and 86.2% amino acid similarity [8]. Hence, we hypothesized that compared to nivolumab, the ability of atezolizumab and avelumab to block the binding of cPD-1/PD-L1, could be based on the conservation of the amino acid residues in the binding site between dog and human. Indeed, alignment of the PD-1 and PD-L1 sequences showed that only two out of six nivolumab/PD-L1 binding residues were conserved in cPD-1 (Figure 2D), while four out of five atezolizumab/avelumab/PD-1 binding residues were conserved in cPD-L1 (Figure 2E) [29].

We also evaluated ipilimumab for binding to cCTLA-4 and a potential functional effect in vitro. Despite specific binding of cCTLA-4 to ipilimumab and the high conservation of the residues (six out of seven residues) [29], we did not detect increased production of cIFN-γ (Figure S2A–D).

### 3.4. Functional Effects of Blockade of cPD-1/PD-L1 Interaction

**Figure 3:**
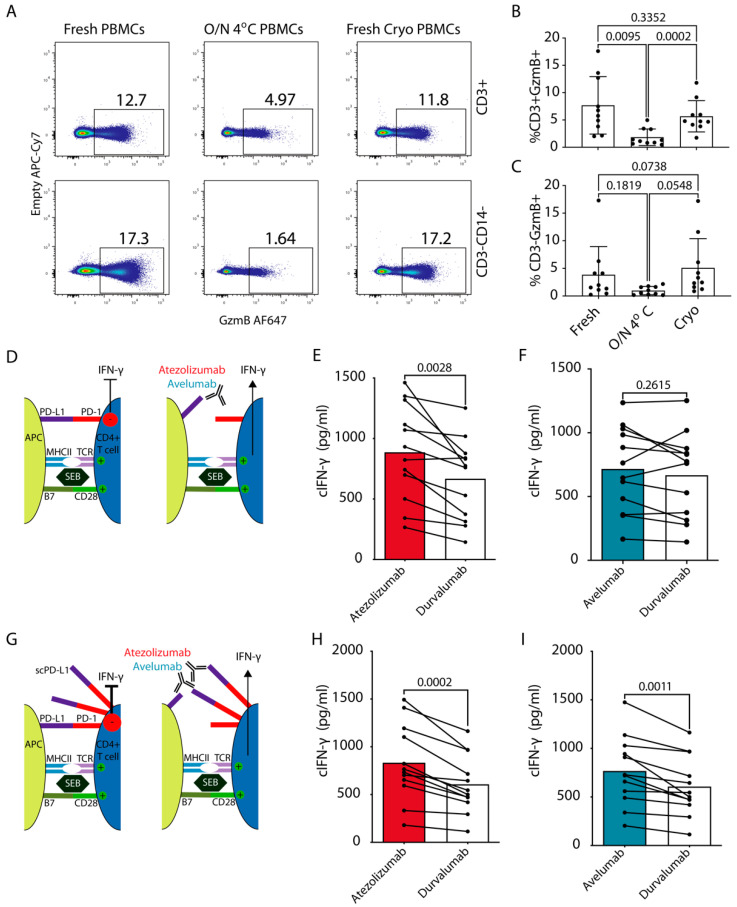
Functional effects of cPD-1/L1 blockade on cIFN-γ production by cPBMCs. (**A**–**C**) Flow cytometric analysis of GzmB production by canine NK and T cells for freshly isolated PBMCs, PBMCs isolated after overnight (O/N) storage of whole blood at 4 °C and freshly isolated, cryopreserved and thawed PBMCs. (**A**) Representative flow cytometric plots depicting gating for T cells (CD3+CD14−) and non-monocytes or T cell fraction, which contains canine NK cells (CD3−CD14−). (**B**,**C**) Bar graphs showing percentages of GzmB producing cells in (**B**) T cells (**C**) NK cell containing fraction (representative plots from three independent experiments, Mean, SEM, Paired One-way ANOVA, *n* = 10). (**D**,**F**) Dog peripheral blood mononuclear cells (*n* = 12, Table 2) were obtained from healthy donors and stimulated with 50 ng/mL of SEB in the presence of 10 μg/mL of atezolizumab or avelumab. Durvalumab was used as a non-binding isotype (hIgG1) control antibody. (**G**–**I**) SEB stimulation as in D–F with addition of 10 μg/mL of scPD-L1 [9] (**D,G**) schematic outlines of the experiments for (**E**,**F**); (**H**,**I**), respectively. For evaluation of cIFN-γ production, the culture supernatant was harvested on day 3, and measured by ELISA. Pooled data from 2 independent experiments (Paired Student’s *t* test with *p* values indicated on the plots).

Before assessing functional benefits of PD-L1 blockade with atezolizumab and avelumab on cPBMCs, we wanted to establish how different timelines of cPBMC isolation affected the functionality of PBMCs. To this end we compared freshly isolated PBMCs, PBMCs isolated after overnight (O/N) storage of whole blood at 4 °C and freshly isolated, cryopreserved and subsequently thawed PBMCs. One of the main observed differences was a significant reduction, in granzyme B production by CD3+ T cells after O/N storage of whole blood at 4 °C compared to freshly isolated PBMCs independent of them being cryopreserved or not (Figure 3A,B). A similar, albeit insignificant trend was also observed for CD3-CD14- fraction containing canine NK cells (Figure 3A,C).

Next, using freshly isolated and cryopreserved PBMCs, we assessed whether the blockade of PD-L1 by atezolizumab or avelumab resulted in functional improvements of cPBMC responses on samples from healthy dogs. For this, we performed an in vitro PBMC stimulation assay using staphylococcal enterotoxin B (SEB) [30], commonly used for validation of ICIs in human PBMCs. IFN-γ production by T cells leads to upregulation of PD-L1 on APCs, which in turn can suppress T cell responses [17,22] (Figure 3D). Atezolizumab induced significantly higher cIFN-γ production by cPBMCs compared to avelumab or durvalumab, the latter was used as a negative non-binding (isotype) control (Figure 3E,F and Figure S3A, Table 2) as all the ICIs in this setup were human IgG1. Atezolizumab and avelumab could still be detected bound to canine APCs (MHCII+CD5−) even after 3 days of culture and stimulation with SEB (Figure S3B).

To further support our findings, we modified the above setup and simulated additional suppression by PD-L1+ cancer cells in the tumor microenvironment by adding 10 μg/mL of soluble (s) cPD-L1 to the activated cPBMCs (Figure 3G and Figure S3C). scPD-L1 binds cPD-1 on T cells and has a suppressive effect [9]. Both atezolizumab and avelumab provided a significant increase in cIFN-γ production compared to durvalumab control (Figure 3H,I). Moreover, only in this setting there was a strong positive correlation between the donor age and the percentage increase in cIFN-γ with atezolizumab compared to durvalumab (Figure S3D). The above results indicate that atezolizumab provides robust functional effect by blocking PD-1 engagement via APC- derived PD-L1, as well as via scPD-L1. On the other hand, avelumab only provided increased cytokine production in presence of additional suppression via scPD-L1. To potentially explain these differences, we compared their binding to canine and human PD-L1 side-by-side over a larger titration range on cIFN-γ activated SCC1 cells and the PD-L1+ human glioma cell line. This showed nearly identical binding profiles of both ICIs to the human tumor cells. This was closely mirrored by atezolizumab on SCC1 cells, while avelumab showed weaker binding to cPD-L1 (Figure S3E).

### 3.5. In Vitro Response of Cancer Patient cPBMCs to Atezolizumab-Mediated PD-L1 Blockade

**Figure 4:**
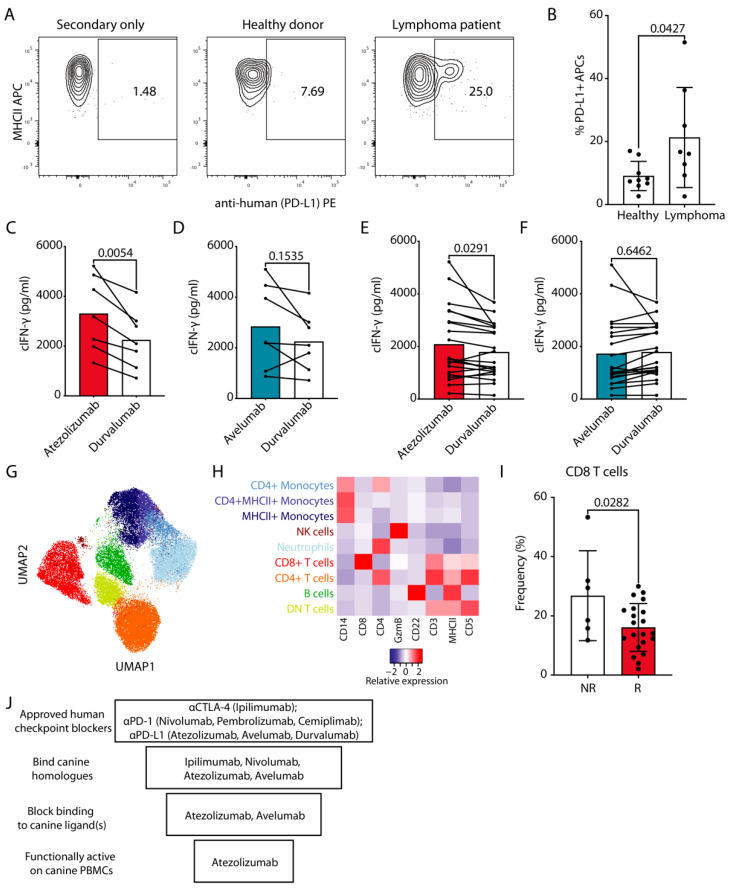
Functional effects of cPD-1/L1 blockade on cIFN-γ production by cancer patient derived cPBMCs. (**A**) Representative staining for PD-L1 on CD5−CD14−MHCII+ APCs with atezolizumab, which was subsequently detected with PE labeled anti-human Fc antibody. (**B**) Pooled data from two independent experiments with each point representing a healthy donor or a lymphoma patient (Mean, SEM, Unpaired Student’s *t* test with *p* value indicated on the plot). (**C**–**F**) cPBMCs from cancer patients (*n* = 27, Table 3) were stimulated and evaluated as in Figure 3D–F. (**C**,**D**) Lymphoma patients, (**E**,**F**) non-lymphoma cancer patients (Table 3). Pooled data from three independent experiments (Paired Student’s *t* test with *p* values indicated on the plots). (**G**,**H**) Cancer patient PBMC samples classified as non-responders (NR, *n* = 6) or responders (R, *n* = 19) as well as healthy donor PBMC samples classified as responders (HD, *n* = 11) (1500 cells/sample) were flow cytometrically analyzed and concatenated; all lymphomas were excluded. (**G**) UMAP clustering analysis with different identified populations colored according to the cluster they were assigned to using the FlowSOM algorithm and manual annotation. (**H**) The heat map represents the normalized median fluorescence intensity (MFI) for respective markers within each cellular population for the nine clusters from concatenated data and was used to annotate the clusters in G. (**I**) Bar graph shows the frequency of CD8+ T cells in the PBMCs of NR vs. R, only T cell lymphomas were excluded. Each point represents a patient (Mean, SEM, Unpaired Student’s *t* test with *p* value indicated on the plot). (**J**) Schematic summary of the evaluation of the effect of human ICIs for dogs.

Next to blockade of PD-L1 expressed on tumors PD-L1 inhibition also on APCs contributes to treatment success [31,32,33]. Lymphoma is one of the indications for which ICI blockade of PD-1/PD-L1 axis is well-established in humans [1]. In the absence of solid tumor samples available to us, we evaluated the levels of PD-L1 on the surface of APCs from lymphoma patients, some of them overtly leukemic (Table 3 and Figure S4). We readily detected PD-L1 on these cells using atezolizumab. The frequency of PD-L1+ cells in the MHCII+CD5-CD14- fraction was significantly higher in lymphoma patients compared to healthy donors (Figure 4A,B), which had also been observed with dog-specific antibodies [12]. Conversely, PBMCs from all 7 tested canine lymphoma patients showed highly significant response to atezolizumab (Figure 4C), whereas avelumab was again ineffective (Figure 4D).

We also evaluated the activity of atezolizumab and avelumab on PBMCs from canine cancer patients with non-lymphoma tumors. For this we repeated the SEB stimulation assay with cPBMCs from dogs with various types of cancer (Table 3). As already observed with PBMCs from healthy donors and lymphoma patients, these samples showed significantly higher cIFN-γ production when the PD-1/PD-L1 axis was blocked with atezolizumab (Figure 4E). Response to avelumab was comparable to non-binding durvalumab control conditions (Figure 4F). We also noted 6 patient samples out of 27, which were either unresponsive to atezolizumab (<5% increase) or showed decreased cIFN-γ production compared to durvalumab (Figure 4E, Table 3).

To compare the atezolizumab responders (R) vs. non-responders (NR) PBMCs we characterized their immune subset composition using our recently developed optimized multicolor immunofluorescence panel [26]. We could clearly detect all major cell lineages in the cancer patient PBMCs, which matched the ones from healthy donors, including monocytes, neutrophils, B cells, NK cells, as well as CD4+, CD8+ or double negative T cells (Figure 4G,H). For a comparison of the composition of R vs. NR PBMCs, we did exclude lymphoma patients to avoid potential leukemic donor samples as confounders for the MHCII+CD5-CD14- fraction. We did not detect significant differences in any major subsets. However, a reduction in CD8+ T cell percentage could be observed in R vs. NR when all cancer samples, including lymphoma patients were analyzed (Figure 4I). PBMC viability between R and NR groups was comparable (S4B).

In summary, using a systematic approach, step-by-step interrogating cross-reactivity, blocking potential and functional effect of FDA-approved ICIs on healthy and cancer patient-derived cPBMCs we evaluated their potential for use in dogs. Out of the 7 candidates, only atezolizumab satisfied all of the above criteria (summarized in Figure 4J) with the majority of cancer patient PBMCs responding with increased cytokine production.

## 4. Discussion

In this study we systematically tested cross-reactivity of seven FDA-approved human ICIs [1] in dogs. This yielded four cross-binding ICIs: ipilimumab, nivolumab, atezolizumab and avelumab. The latter two also showed blocking efficacy of canine PD-1/PD-L1 interaction. Atezolizumab provided robust functional benefits when used in cPBMC activation in vitro assays and significantly improved cIFN-γ production in samples from healthy donors and cancer patients alike. In contrast, avelumab only led to improvement of cytokine production in presence of additional scPD-L1. Moreover, atezolizumab had a similar binding profile to cPD-L1 when compared to hPD-L1, while avelumab saturated at a higher concentration (Figure S3E). Another potential explanation for the observed differences could be the Fc effector function of avelumab: unlike atezolizumab and durvalumab, avelumab is the only PD-L1 blocker with a non-silenced Fc and this may influence its efficacy in certain types of cancer [34]. PD-L1 is known to be expressed on both tumor cells and APCs [32,33]. The SEB stimulation assay requires APC-derived MHCII binding to CD4 T cell receptor for effective CD4+ T cell activation and IFN-γ production [30]. In this assay, avelumab may promote antibody dependent cellular cytotoxicity (ADCC) of PD-L1+ APCs through its non-silenced Fc, potentially dampening APC derived T cell activation. However, we could not detect any functional benefits of avelumab testing cancer-patient derived PBMCs, even in lymphoma samples of leukemic patients which showed robust PD-L1 expression. Of note, the primary effector cells of ADCC—NK cells—are still relatively poorly described in dogs and it is also largely unknown which IgG subclasses potently mediate ADCC [35,36,37].

In general, canine Fc gamma receptors (FcγRs) and neonatal Fc receptor (FcRn) appear to be compatible between humans and dogs [35,38]. Dogs have previously been used to assess pharmacokinetics of human(ized) antibodies [38]. Also, the appearance of anti-drug antibodies (ADA) against biologics results in noticeable decreases in drug concentrations over time, which was not observed [38]. Moreover, a canine study with the humanized anti-CCR4 antibody mogamulizumab showed depletion of regulatory T cells that remained consistent throughout the study, indicating that the potential development of ADA did not impair functionality for extended treatment timeframes [39]. In a recent study testing chimeric and caninized anti-PD-1 antibodies for treatment of dog cancer patients, the appearance of ADAs against the caninized version did not result in the reduction of the antibody availability in circulation for at least two weeks [16]. In neither case were severe side-effects detected. This points at the need to further establish the role of ADAs in dogs and again highlights that the success of a therapeutic antibody is determined not only by its blocking ability, but also by a plethora of important developability parameters, which are especially enriched in the clinically approved ICIs, such as atezolizumab [20].

To date very few PBMC biomarkers are known that predict the efficacy of ICIs in human cancers [31]. However, several studies attempted to evaluate such potential biomarkers retrospectively. One of those has shown that increased percentage of classical monocytes and decreased fraction of T cells in responder compared to non-responder PBMCs could predict the efficacy of PD-1/PD-L1 axis blockade [40]. While we could not detect any differences in the monocyte compartment, we noticed a significant reduction in CD8+ T cells in R vs. NR patient derived cPBMCs. An interventional trial using atezolizumab in canine cancer patients and a detailed analysis of pre-treatment PBMCs, as well as assessment of the intratumoral PD-L1 expression would be necessary to further evaluate the predictive value of our observations.

In light of our results, we expect that efficacy tests in cancer-bearing dogs are feasible with atezolizumab. Our in vitro results and in vivo studies using canine specific chimeric antibodies targeting the PD-1/PD-L1 axis [16,17] suggest that also in dogs, not all patients will benefit from atezolizumab monotherapy. In humans, it is well-established that only certain types of cancers are responsive to ICIs alone [41]. Therefore, various combination immunotherapies against cancer have been proposed [42,43,44]. One of the many modalities tested in combination with ICIs is the highly potent immune-activating cytokine interleukin 12 (IL-12) [44,45]. Human IL-12 is cross functional on dog immune cells and has been tested as electrogenetherapy [46] as immunocytokine (NHS-IL-12 [47]) or armed oncolytic virus [48] in dogs. Preclinical mouse studies have shown synergistic effects of PD-1/PD-L1 blockade with murine surrogates for NHS-IL-12 [49]. We believe that our study calls for an evaluation of atezolizumab in canine cancer patients alone or in combination with potent immunoactivating cytokines such as IL-12. Overall, our results show robust activity of atezolizumab on cPBMCs in the context of healthy and cancer patients. In our study, cancer patients and healthy donors had a substantial difference in age distribution. When we explored the relationship between age and functional responses to atezolizumab we observed a positive correlation (R^2^ = 0.77) in the extent of response with increasing age in the SEB assay plus additional scPD-L1 (Figure S3D). Moreover, atezolizumab was particularly efficacious on PBMCs from lymphoma patients, which expressed higher levels of PD-L1 and potentially also contained the actual cancer cells in some cases. In human Hodgkin’s lymphoma (HL), there is a common amplification of chromosome 9p24.1, which encodes PD-L1 [50]. This probably makes HL highly responsive to ICIs [51,52]. On the other hand, in T cell lymphomas PD-1 can play a tumor suppressive role [53] and use of ICIs can lead to progression of the disease [54]. Hence, the use of ICIs in this type of lymphoma requires careful evaluation of the disease immune landscape to identify subsets of patients who likely benefit from ICIs.

Testing of human cancer immunotherapies in dogs could yield profound synergistic effects for the fields of human and canine oncology. Dog patients with cancer can benefit from immunotherapies for which the development would have been too costly for the dog alone. Vice versa, data from canine trials with cross-reactive therapies would yield insights on tolerability, efficacy, potential biomarkers and combination therapies for their clinical translation. However, it is also worth mentioning that while the seven tested ICIs have all been clinically approved for humans, only one showed cross-functionality on canine PBMCs, which could restrict generalization of such an approach. Similar to humans, dogs show a remarkable genetic diversity as overall species, and at the same time, pure breeds represent a highly homogenous genetic makeup [4]. Testing the efficacy of checkpoint inhibition across different pure breeds could give valuable insights to further understand mechanisms of response (or lack thereof) in human patients.

We are aware that the here described assessment of the efficacy of ICIs using ex vivo PBMC assays has limitations also in the human setting [22,23]. In some cancers the suppressive state of patient PBMCs can reach levels comparable to the tumor microenvironment, however, in most cases it is likely that local immunosuppression will be substantially higher. While we attempted to simulate additional immunosuppression using scPD-L1, it could be interesting to assess the effect of PD-L1 blockade on cells isolated from tumor tissue or directly on tumor explants. Expanding on the here-presented stepwise approach with commercially available reagents, human ICIs can be tested for their suitability for conducting efficacy testing in dogs, using the described mix of in vitro binding assays and cell-based assays. Subsequently, the data from those trials could help refine dose estimations and regimens for first-in-human or combination studies. Data from such trials would also provide a rationale for further implementation of ICIs in veterinary oncology.

## 5. Conclusions

The PD-1/PD-L1 axis is currently the most frequently targeted immune checkpoint in immunooncology and the number one choice for combinatorial immunotherapeutic approaches [3]. In vitro and in vivo studies report increased PD-1/PD-L1 expression in canine cancers and successful targeting of these checkpoints in patient dogs [16,17]. Yet, commercially available antibodies for veterinary medicine are missing. Our in vitro and ex vivo data using healthy and cancer patient derived canine PBMCs underscore cross-functionality of atezolizumab, an approved PD-L1 blocker, between humans and dogs and provide a rationale for clinical testing of atezolizumab in veterinary oncology. Moreover, we think that dogs can provide a complementary tool and a bridge from murine cancer models to clinics. Finally, we hope our study will encourage first in dog reverse translational efforts using (combination) immunotherapies in companion dog tumor patients with potential to benefit both veterinary and human medicine.

## Figures and Tables

**Table 1:** List and details of flow cytometric antibodies used in this study.

#	Specificity	Fluorochrome	Clone	Dilution	Staining	Manufacturer	Product	Target	Host
1	CD45	eFluor 450	YKIX716.13	1:50	Surface	Thermo Fisher	48-5450-41	Dog	Rat
2	Dead cells	Aqua	N/A	1:500	Surface	Biolegend	423102	N/A	N/A
3	CD25	SB600	P4A10	1:50	Surface	Thermo Fisher	63-0250-41	Dog	Mouse
4	CD4	SB645	YKIX302.9	1:10	Surface	Thermo Fisher	64-5040-42	Dog	Rat
5	CD8	SB702	YCATE55.9	1:25	Surface	Thermo Fisher	67-5080-42	Dog	Rat
6	CD14	BV785	M5E2	1:100	Surface	Biolegend	301840	Human	Mouse
7	CD3	FITC	CA17.2A12	1:10	Surface	BioRad	MCA1774F	Dog	Mouse
8	Eomes	PerCP-eFluor710	WD1928	1:100	IC	Thermo Fisher	46-4877-42	Human	Mouse
9	CD22	PE	RFB-4	1:50	Surface	Thermo Fisher	MHCD2204	Human	Mouse
10	Granzyme B	PE-CF594	GB11	1:1600	IC	BD	562462	Human	Mouse
11	Granzyme B	Alexa Fluor 647	GB11	1:800	IC	BD	560212	Human	Mouse
12	FoxP3	PE-Cy7	FJK-16s	1:800	IC	Thermo Fisher	25-5773-80	Human	Rat
13	MHCII	APC	YKIX334.2	1:25	Surface	Thermo Fisher	17-5909-42	Dog	Rat
14	Ki67	Alexa Fluor 700	SolA15	1:6400	IC	Thermo Fisher	56-5698-82	Rat	Rat
15	CD5	APC-eFluor780	YKIX322.3	1:50	Surface	Thermo Fisher	47-5050-42	Dog	Rat
16	human Fc	PE	HP6017	1:200	Secondary	Biolegend	409304	Human	Mouse

**Table 2:** Characteristics of healthy donors used in the study. Responders (R), non-responders (NR) to atezolizumab.

#	Sex/Castrated	Age	Breed	R/NR	Atezolizumab/Durvalumab Response (%)	Atezolizumab/ Durvalumab Response with scPD-L1 (%)
1	Female	5	Mixed Breed	NR	−1.90	21.01
2	Female spayed	3	Berger Blanc Suisse	R	5.08	23.23
3	Female	7	Barsoi	R	22.64	54.51
4	Female	9	Flat Coated Retriever	R	88.70	64.77
5	Female spayed	6	Mixed breed	R	59.71	37.99
6	Female	7	Continental Bulldog	R	7.89	54.07
7	Female	7	Boxer	R	50.31	41.58
8	Male	4	Golden Retriever	R	98.52	40.46
9	Male castrated	6	Vizla	R	34.16	56.02
10	Female spayed	4	Labrador Retriever	R	22.69	13.16
11	Female	8	Boxer	R	84.60	57.57
12	Female	1	Golden Retriever	R	32.06	9.09

**Table 3:** Characteristics of cancer patients used in the study. Responders (R), non-responder (NR) to atezolizumab.

#	Sex/Castrated	Age	Breed	Diagnosis	Leukaemic	R/NR	Atezolizumab/ Durvalumab Response (%)
1	Female spayed	10	Czechoslovakian Wolfdog	Mast cell tumor		R	19.09
2	Female	9	German Shepherd	Oral malignant melanoma		R	17.96
3	Male castrated	10	Mixed Breed	Malignant B cell lymphoma		R	51.77
4	Female spayed	8	Labrador Retriever	Mast cell tumor		NR	−24.41
5	Female spayed	12	Mixed Breed	Mast cell tumor, meningioma		R	15.79
6	Female	3	German Boxer	Mast cell tumor		R	28.01
7	Female spayed	11	Mixed Breed	Anal sac adenocarcinoma		NR	−29.37
8	Female spayed	12	French Bulldog	Mast cell tumor		NR	−9.66
9	Male castrated	8	Mixed Breed	Prostate carcinoma		R	24.21
10	Male castrated	14	Mixed Breed	Malignant B cell lymphoma		R	74.79
11	Male castrated	8	Golden Retriever	Mast cell tumor		R	21.80
12	Male castrated	11	Beagle	Mast cell tumor		R	7.99
13	Male	6	Bernese Mountain Dog	Malignant B cell lymphoma		R	41.88
14	Female	10	Golden Retriever	Soft tissue sarcoma		R	8.65
15	Female spayed	9	Mixed Breed	Mast cell tumor		R	37.05
16	Female spayed	12	Mixed Breed	Hepatocellular carcinoma		NR	−12.41
17	MK	11	Italian Segugio	Malignant T cell lymphoma	x	R	16.76
18	Female spayed	10	Mixed Breed	Sinonasal carcinoma, pulmonary carcinoma		R	52.09
19	Female spayed	8	Mixed Breed	Meningioma		R	14.59
20	Male	6	Rhodesian Ridgeback	Mast cell tumor		R	40.02
21	Male castrated	10	Golden Retriever	Oral malignant melanoma		NR	2.89
22	Female spayed	6	Rhodesian Ridgeback	Malignant T cell lymphoma		R	85.73
23	Male castrated	5	Bernese Mountain Dog	Malignant B cell lymphoma		R	26.58
24	Male	9	Labrador Retriever	Mast cell tumor		R	8.88
25	Female spayed	13	Hungarian Viszla	Sinonasal carcinoma, pulmonary carcinoma		NR	−15.54
26	Male	12	Golden Retriever	Malignant lymphoma	x	R	86.67
27	Male castrated	8	Golden Retriever	Mast cell tumor		R	16.46

## Data Availability

The data presented in this study are available on request from the corresponding author.

## Supplementary Materials

The following are available online at https://www.mdpi.com/2072-6694/13/4/785/s1, Figure S1: PD-1 and PD-L1 expression on cPBMCs and canine cell lines, Figure S2: Evaluation of Ipilimumab, Figure S3: Durvalumab vs. isotype ctrl, binding of PD-L1 blockers to canine APCs, Suppression of the SEB assay with scPD-L1 and age vs. response correlation, binding comparison of atezolizumab and avelumab to human and canine cells, Figure S4: Flow cytometric gating strategy for canine APC and viability comparison of NR vs. R.
