# Supplementary material for: Cross-Reactivity and Functionality of Approved Human Immune Checkpoint Blockers in Dogs
Source: Cancers (Basel). 2021 Feb 13;13(4):785. doi: 10.3390/cancers13040785 (PMC7918463; doi:10.3390/cancers13040785)
Supplement: Supplementary file 1 [file cancers-13-00785-s001.pdf]

cancers

Supplementary Materials

Cross-Reactivity and Functionality of Approved Human Immune Checkpoint Blockers in Dogs

Stanislav Pantelyushin, Elisabeth Ranninger, Diego Guerrera, Gregor Hutter, Caroline Maake, Enni Markkanen, Regula Bettschart-Wolfensberger, Carla Rohrer-Bley, Heinz Läubli and Johannes vom Berg

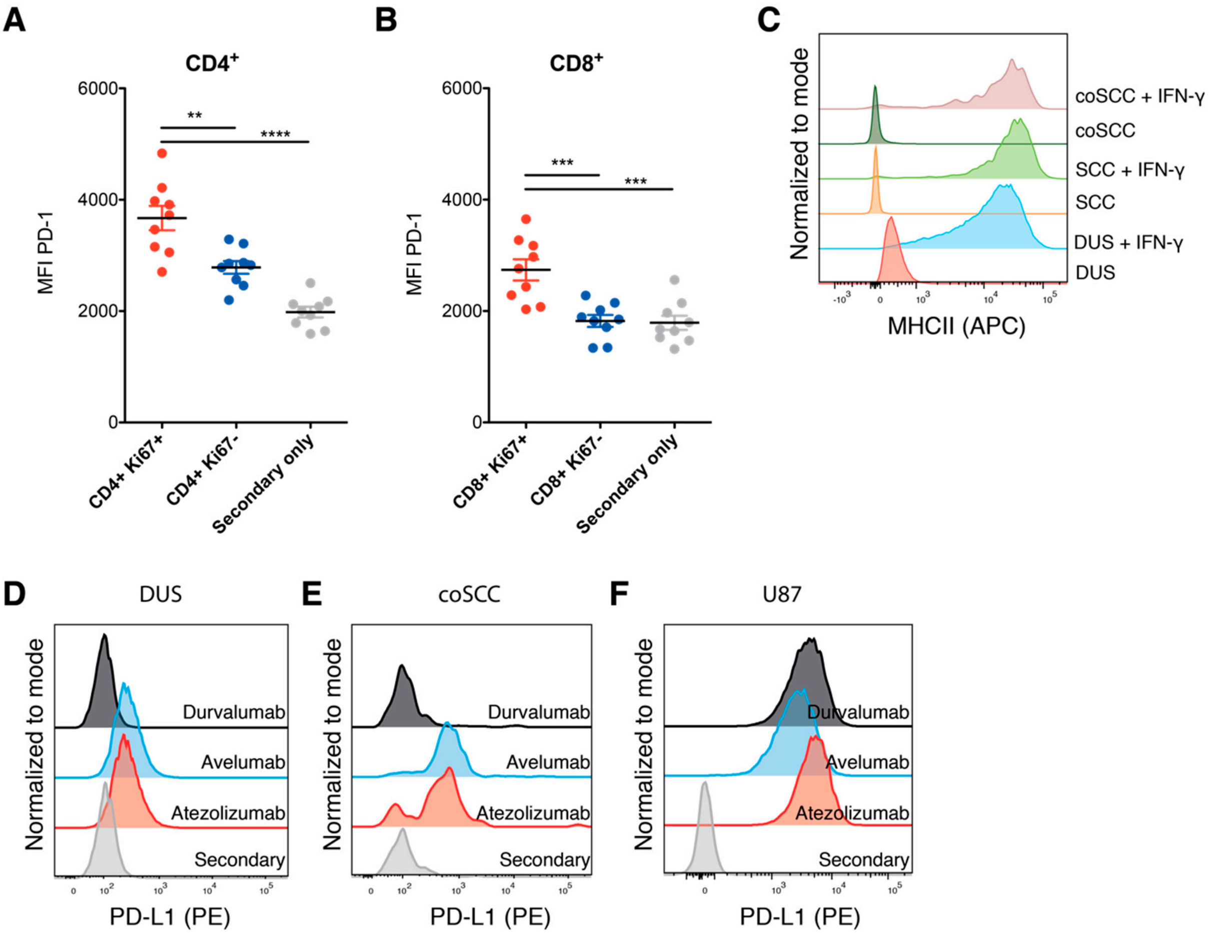

Figure S1. (**A**,**B**) Scatter plots show MFIs of Nivolumab on Ki67+ vs. Ki67- cells. Canine PBMCs from 9 healthy donors were stimulated with 2.5 µg/mL of ConA for 48 h. (Mean and SEM, One-way Repeated measures ANOVA with Tukey comparison of all pairs of columns, ** $p < 0.01$, *** $p < 0.001$, **** $p < 0.0001$, significant differences indicated). All pregated on CD45+ live single cells. (**C**–**F**) Representative flow cytometric histograms showing (**C**) upregulation of canine MHCII on canine cell lines incubated with 20 ng/mL of cIFN-γ for 48 h. (**D**,**E**) binding of atezolizumab, avelumab and durvalumab to cPD-L1 on canine cell lines after 48 h stimulation with 20 ng/mL of cIFN-γ (**D**) DUS cell line (**E**) coSCC cell line. (**F**) binding of atezolizumab, avelumab and durvalumab to a human glioma cell line U87, which constitutively expresses PD-L1. All pregated on live single cells.

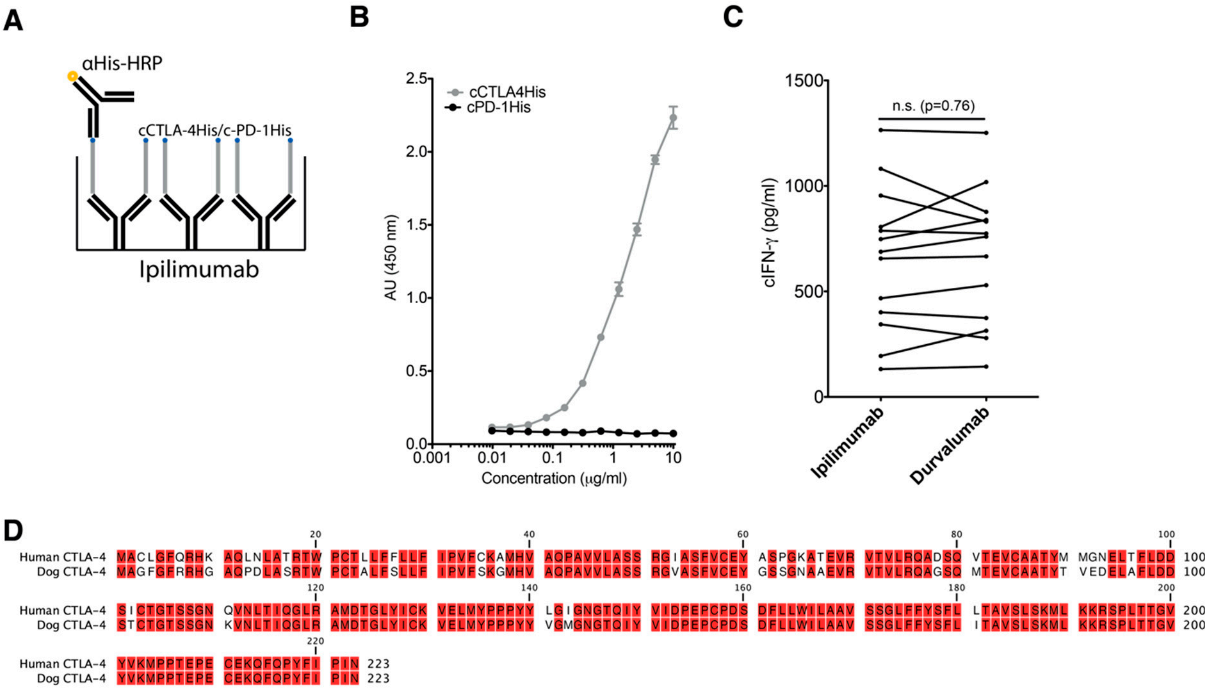

Figure 2. (**A,B**) binding of cCTLA-4His vs. cPD-1His to plate bound ipilimumab (**A**) schematic representation of the experiment (**B**) ELISA measured curves showing detection of His tag bound ligands to ipilimumab. (**C**) Sequence alignments of amino acid sequences of canine and human CTLA-4, with black arrows depicting hotspot residues necessary for their binding and blocking. (**D**) Comparing 10 μg/mL treatment with ipilimumab against non-binding durvalumab, after polyclonal activation with 50 ng/mL of SEB for 72 h. ELISA measurement of supernatant secreted cIFN-γ by cPBMCs from 12 healthy donors (Mean and SEM Student two-tailed paired *t* test * $p < 0.05$).

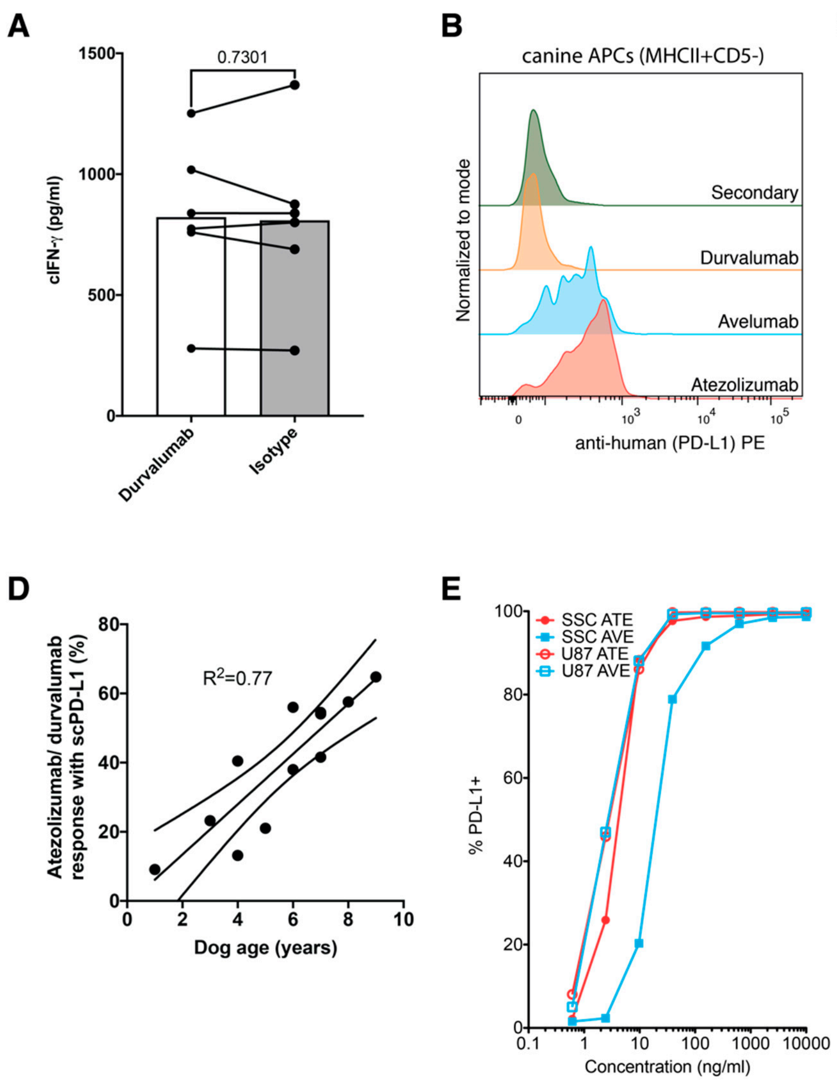

Figure S3. (**A**) Bar graphs showing cPBMC production of cIFN-γ stimulated with 50 ng/mL of SEB in the presence of 10 µg/mL of durvalumab or human IgG1 isotype control (**B**) Representative flow cytometric histograms showing PD-L1 ICIs bound to cPD-L1 on APCs after experimental setup as used in Figure 3D–F. Pregated on live, single, MHCII+CD5- cells (representative plot of 2 independent experiments, *n* = 12). (**C**,**D**) Suppression of cPBMC responses in 12 healthy donors (Table 1) to 50 ng/mL of SEB with the addition of 10 µg/mL of scPD-L1 after 72 h (**C**) as quantified by production of cIFN-γ. (Mean and SEM, Student two-tailed paired *t* test). (**D**) Graph plotting % increase in cIFN-γ production atezolizumab/durvalumab against the age of the donors (pooled data from two independent experiments, *n* = 12). (**E**) Comparison and quantification of flow cytometric binding of Atezolizumab and Avelumab to U87 vs. 48 h cIFN-γ stimulated SCC1 cell line (Representative plot of two independent experiments).

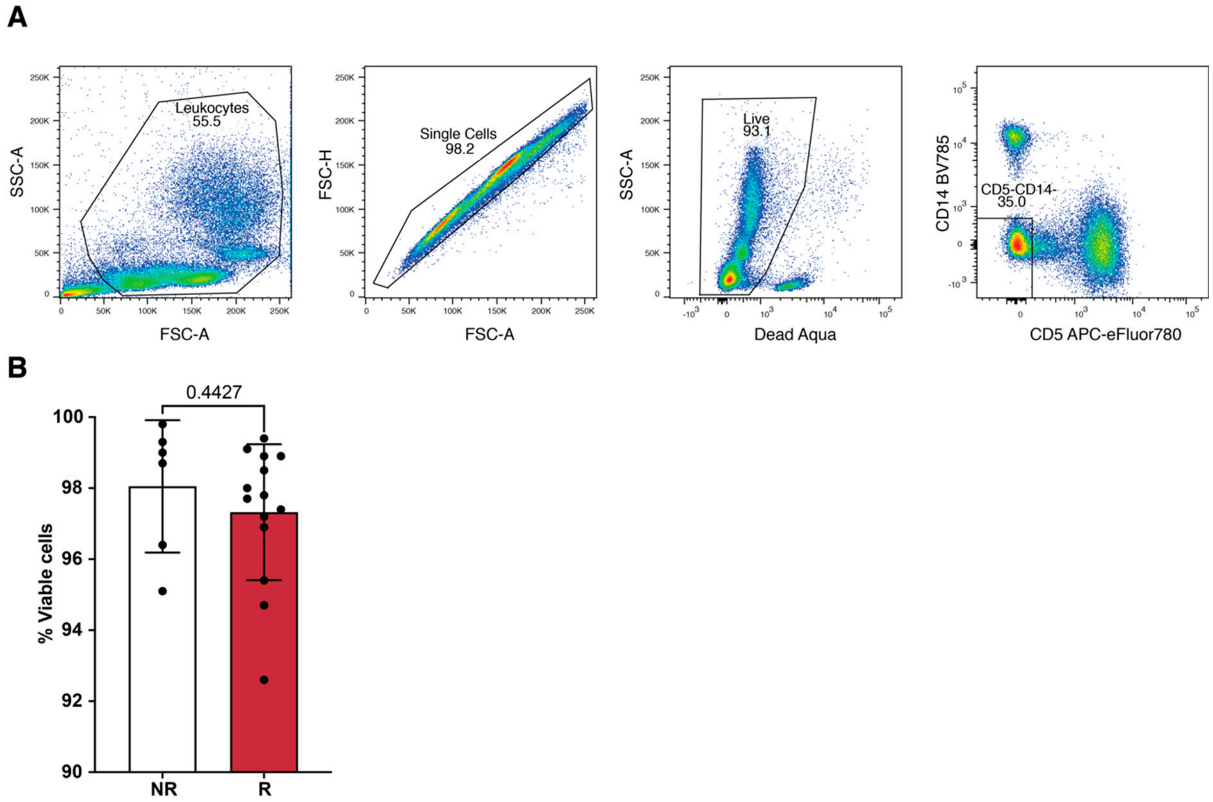

Figure S4. (**A**) Representative example of gating strategy for flow cytometric analysis of PD-L1 expression on canine APCs. (**B**) Bar graph plotting distribution of % viable cells in the atezolizumab NR and R groups from Figures 4E and I (pregated on single cells, mean and SEM Student two tailed unpaired *t* test).
